# The Use of CellCollector Assay to Detect Free Cancer Cells in the Peritoneal Cavity of Colorectal Cancer Patients: An Experimental Study

**DOI:** 10.1002/cam4.70378

**Published:** 2024-11-06

**Authors:** Yudi Wu, Fangxun He, Liang Liu, Wei Jiang, Jiao Deng, Yujie Zhang, Zhixin Cao, Xiangshang Xu, Jianping Gong

**Affiliations:** ^1^ Department of Gastrointestinal Surgery Tongji Hospital, Huazhong University of Science and Technology Wuhan China; ^2^ GI Cancer Research Institute Tongji Hospital, Huazhong University of Science and Technology Wuhan China

**Keywords:** CellCollector, colorectal cancer, intraperitoneal free cancer cells, real‐time PCR

## Abstract

**Background:**

Colorectal cancer (CRC) is associated with high incidence and mortality rates globally. The presence of intraperitoneal free cancer cells (IFCCs) is recognized as an independent prognostic factor for CRC patients. However, a clinical gold standard for IFCCs detection is lacking. The GILUPI CellCollector has demonstrated high sensitivity and specificity in detecting free cancer cells, yet its application for CRC IFCCs detection remains unreported.

**Methods:**

We selected CRC and normal cell lines to evaluate the CellCollector's ability to detect tumor cells. A total of 70 CRC patients and 17 patients with benign disease undergoing laparoscopic procedures were investigated. Peritoneal lavage fluid was collected pre‐ and post‐operation, and both real‐time PCR (CEA mRNA) and CellCollector detection were performed. We compared the sensitivity and specificity of these two methods.

**Results:**

CellCollector can distinguish well between CRC and normal cells in cell line experiments. CellCollector detects IFCCs better than real‐time PCR (CEA) in CRC patients in different TNM Stages. The sensitivity of CellCollector was higher than that of real‐time PCR (84.6% vs. 48.4%), and the specificity of CellCollector was also higher than real‐time PCR (79.1% vs. 60.4%). There was no significant difference in the results of IFCCs detected by CellCollector before and after total mesorectal excision (TME) or complete mesocolic excision (CME) radical colorectomy (*p* > 0.05), but there was a significant difference in real‐time PCR detection (*p* < 0.05).

**Conclusions:**

The CellCollector demonstrates superior sensitivity and specificity compared to real‐time PCR for detecting IFCCs in CRC patients, suggesting its potential as a clinical tool for IFCCs detection.

**Trial Registration:**

ClinicalTrials.gov identifier: NCT01978444

## 1 Introduction

Colorectal cancer (CRC) ranks third in incidence and second in mortality among cancers globally [1]. Despite radical tumor resection, approximately 10%–30% of patients experience cancer recurrence due to shedding of cancer cells from the primary site [2, 3, 4, 5]. Peritoneal implantation is a critical metastatic pathway in gastrointestinal cancers. Detecting IFCCs in intraoperative lavage fluid can indicate intraperitoneal cancer spread [6, 7]. Advances in treatment methods, including cytoreductive surgery (CRS) and hyperthermic intraperitoneal chemotherapy (HIPEC), have shown promising outcomes, particularly for gastrointestinal malignancies [8, 9]. Thus, accurate assays for IFCCs detection are essential.

IFCCs can be identified in peritoneal lavage fluid, with pre‐ and postoperative positivity serving as independent prognostic factors for CRC patients [6, 7]. Currently, no gold standard exists for IFCCs detection. Traditional cytology is widely used due to its 100% specificity, [10] but it has a low positive detection rate (often below 10%) [11, 12, 13, 14, 15, 16, 17, 18, 19], limiting its clinical utility. Which limits its clinical application. To enhance diagnostic accuracy, immunocytochemistry (ICC) has been employed with various monoclonal antibodies, yielding positive rates of 20%–30% for IFCCs. However, ICC's specificity is suboptimal [20], particularly in stage I/II patients [21].

Real‐time polymerase chain reaction (qPCR) allows for precise quantification of DNA amplification through fluorescent detection [22]. This method's high sensitivity enables it to detect target DNA concentrations over a vast dynamic range, with common targets being CEA and CK20 in peritoneal washings from CRC patients [17]. While CEA correlates with prognosis, qPCR's high sensitivity can lead to false positives, particularly in benign cases, due to elevated CEA and CK20 expression in inflammatory non‐tumor cells [23]. In addition, some patients with benign diseases have shown positive cancer biomarkers [17]. This low specificity may be related to the high expression of CEA and CK20 in non‐tumor cells in the inflammatory environment [24]. This lack of specificity may result in unnecessary postoperative treatments and patient anxiety.

Most studies on IFCCs detection have focused on preoperative lavage, with few examining postoperative samples. Current literature indicates variability in detection rates: one study reported a decrease to 3% postoperative detection, while another noted an increase to 20% [17, 25]. The inconsistency in pre‐ and postoperative qPCR results raises questions about reproducibility and stability, making it crucial to assess IFCCs positivity around surgical intervention as a potential prognostic factor [10]. Emphasis is increasingly placed on developing assays that offer improved sensitivity and specificity.

The GILUPI CellCollector, an established medical device, utilizes antibodies against epithelial adhesion molecules (EpCAM) to capture circulating tumor cells (CTCs) from peripheral blood [26, 27, 28]. Its design allows approximately 1 L of blood to flow through the functional area, enhancing CTC capture while ensuring high purity of isolated cells (> 90%) [29]. However, its application for detecting IFCCs in peritoneal lavage fluid remains unexplored.

**FIGURE 1:**
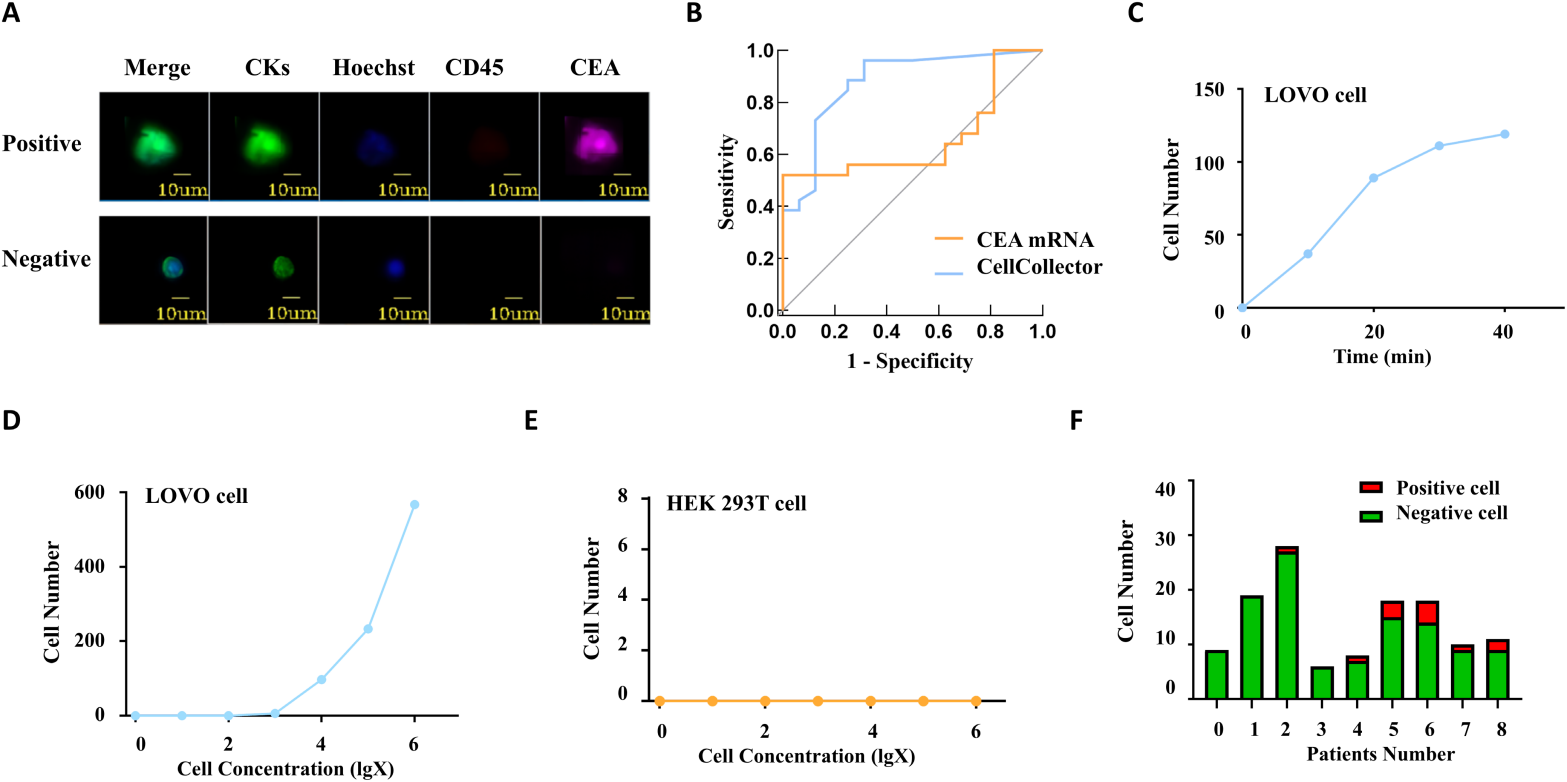
CellCollector assay in detecting CRC cells. (A) The way Cellcollector is used to distinguish CRC cells from other cells. (B) ROC curves of CellCollector and real‐time PCR assay in detection of CRC cells. The area under the curve of CellCollector and real‐time PCR were 0.876 and 0.671, respectively. (C) Relationship between the number of tumor cells captured by CellCollector and the detection time. The colon cancer cell line Lovo cell concentration was 5 × 10^4^ cells/mL. (D) Relationship between the number of tumor cells captured by CellCollector and cell concentration. The colon cancer cell line Lovo cell concentration is 0, 10^1^, 10^2^, 10^3^, 10^4^, 10^5^, 10^6^ cells/mL. (E) Verification of CellCollector's ability to capture cells in HEK‐293 T cells. The cell concentration was 5 × 10^4^ cells/mL. (F) Normal epithelial cells and positive cell capture in abdominal lavage fluid from clinical patients. No. 1–4, patients with benign disease; No. 5–9, patients with colorectal cancer.

Based on the working principle of CellCollector, it may also be used to detect IFCCs (Figure 1A). This study aims to evaluate the efficacy of the CellCollector and qPCR (for CEA expression) in detecting IFCCs in the abdominal lavage fluid of CRC patients. We hypothesize that CellCollector technology may offer superior sensitivity and specificity compared to conventional qPCR, ultimately enhancing prognostic assessment and guiding treatment decisions for CRC patients.

## 2 Materials and Methods

### 2.1 Cell Lines

Human colon cancer cell lines Lovo, SW480 and human normal embryonic kidney cells‐HEK 293 T cells were purchased from the IBS Cell Bank of Fudan University and the Cell Bank of Shanghai Institute, Shanghai, China. Cells were cultured in DMEM (Gibco BRL, supplemented with 10% FBS). Cells were grown for no more than 20 passages for any experiment.

### 2.2 Patients and Specimens

All patients were extensively informed and gave written consent to the investigation. The study was approved by the Ethics Committee of Tongji Hospital, Tongji Medical College, Huazhong University of Science and Technology (Wuhan, China) (TJ‐IRB20230215). Seventy patients with colorectal cancer and 17 patients with benign disease who underwent surgery in the Department of Gastrointestinal Surgery at Tongji Hospital from April 2023 to August 2023 were investigated. Patients with prior abdominal surgery or other malignant diseases were excluded from the study. No preoperative chemotherapy or radiotherapy was performed in the included patients. All patients underwent laparoscopic surgery.

The collection of exfoliated cells by abdominal lavage was performed in two stages, the first stage was performed after laparoscopic exploration but before removal of the tumor, and the second stage was performed towards the end of the surgery after the tumor had been removed. All colorectal cancer operations were performed in accordance with standard total mesorectal excision (TME) or complete mesocolic excision (CME) [30, 31] and the “no touch cancer” principle was followed during the operation to minimize the spread of cancer cells from medical sources. When the peritoneal lavage fluid was collected, we injected 200 mL of saline through the laparoscope at the tumor site for flushing and aspirated at least 150 mL for testing. All samples were immediately transferred from the operating room to the laboratory for next processing. Each washout sample was divided equally into three for cytology, real‐time PCR and CellCollector assays.

### 2.3 CellCollector Assay

For colon cancer cell lines, the CellCollector probes were placed in 1.5 mL of saline at multiple dilutions (10^2^–10^6^ cells/mL), ensuring complete submersion of the functional area. After incubation for half an hour on a shaker at 37°C, the probe was carefully removed, washed three times in PBS solution and then fixed in methanol for 5 min, followed by immunofluorescence staining in an epithelial cell autostainer using a kit; after the staining was completed, the probe was placed in a stained cell image scanning system for image scanning and positive cell interpretation. CellCollector was assayed at different concentrations of cell acquisition. A concentration of 5 × 10^4^ cells/mL was selected to calculate the capture volume using CellCollector probes incubated for 10, 20, 30, and 40 min, respectively.

For clinical washout samples, the precipitate was taken after centrifugation and dissolved in 1.5 mL of saline, and the subsequent assay steps were the same as for the cell lines.

### 2.4 Real‐Time PCR Assay

Each sample was centrifuged at 1000 rpm for 5 min, and then total RNA was extracted by the TRIzol method (Invitrogen, Carlsbad, CA) according to the procedure provided by the manufacturer. The total amount and purity of total RNA were assessed by measuring the optical density ratio at 260/280 nm spectrophotometrically. Extracted total RNA samples were stored in a −80°C refrigerator. After denaturation in DEPC‐treated water at 70°C for 10 min, 1 mg of total RNA was used for cDNA synthesis using cDNA Synthesis Mix (Takara, Tokyo).

Gene sequences were obtained from the NCBI database. Oligonucleotide primers for CEA and β‐actin were chosen with the assistance of the NCBI primer blast program. The primer sequences used throughout this study are described in (Table S1). Oligonucleotide primers were purchased from Invitrogen.

Real‐time PCR was performed in the QuantStudio 6&7 Real‐Time PCR System (Appliedbiosystems, USA) with optimized PCR conditions. The reaction was carried out in a 384‐well plate using TB Green Supermix 2 (Takara, Tokyo). All assays included a negative control and were replicated three times. The relative expression of β‐actin was used for standardizing the reaction. The thermal cycling conditions comprised an initial denaturation step at 95°C for 3 min, followed by 40 cycles at 95°C for 10 s and 60°C for 30 s.

Quantitative values are obtained from the *C*_t_ number at which the increase in signal associated with the exponential growth of PCR products starts to be detected. Target genes (CEA) amplification was compared with simultaneous amplification of an endogenous reference gene (β‐actin), and each sample was normalized based on its β‐actin content. The target genes CEA were tested for expression in tenfold serial dilutions (10^2^–10^6^ cells/mL) of colon cancer cell lines (SW480, Lovo). A normal human cell line (HEK 293 T) was used as a negative control.

For data analysis, receiver‐operating characteristic (ROC) curves were used to compare the accuracy of positive cell number/total capture cells number ratio in CellCollector assay, CEA/β‐actin ratio and determine the cut‐off value by plotting sensitivity/ specificity pairs for the RNA ratio (Figure 1B). The clinical value of CellCollector, CEA detection was assessed based on the diagnostic data from patients with positive cytology made at laparoscopy and from patients of the negative control group (the patients information could be seen in Table S2). The cut‐off value for CellCollector was defined as 0.07, and for CEA was defined as 0.00001 (CEA/β‐actin ratio).

### 2.5 Statistics

A cross‐tabulation analysis of CellCollector and real‐time PCR analysis was performed by the chi‐square test for trend or Fisher's exact test. The Wilcoxon test was chosen for paired information where the distribution is not positively distributed. A *p*‐value of 0.05 was considered as statistically significant. In the absence of a gold standard, the sensitivity and specificity of the two assays were evaluated using the software package DTAXG in R language.

## 3 Results

### 3.1 CellCollector Can Distinguish Between Tumor Cells and Normal Cells

The CellCollector assay demonstrated a clear capacity to distinguish between tumor cells and normal cells. As expected, the number of cells captured from colon cancer cell lines increased proportionally with the initial cell concentration. In contrast, no positive cell captures were observed in the HEK 293 T cell line. Furthermore, the capture efficiency of the CellCollector assay on colon cancer cell lines increased over time, peaking at 30 min post‐initiation, after which a plateau was reached with no further increase in capture (Figure 1C–E).

**FIGURE 2:**
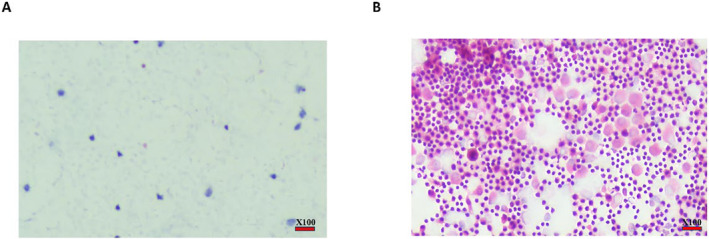
Cytology to detect IFCCs. (A) Cytology of peritoneal lavage fluid from patients with benign disease, tumor cells could not be detected in the ascites, and a few mesenchymal and inflammatory cells were seen. (B) Cytology of peritoneal lavage fluid from positive patients with colorectal cancer, many tumor cells, and inflammatory cells can be detected in the ascites. ×100, the scale bar means 100 times magnification.

In the preliminary assessment, we selected four patients with benign conditions and five patients diagnosed with colorectal cancer (cT4 stage) for evaluation. Abdominal lavage fluid was collected from these patients and analyzed using the CellCollector assay. The cytological examination of the peritoneal lavage fluid is presented in Figure 2. Among the four patients with benign diseases, only one positive cell was detected in patient #3, resulting in a specificity of 75% for the CellCollector technique. Conversely, all five patients with colorectal cancer exhibited positive cell detection in their abdominal lavage fluid, yielding a sensitivity of 100% (Figure 1F, Table S3).

### 3.2 CellCollector Detects IFCCs Better Than Real‐Time PCR (CEA)

In a study involving 70 CRC patients, the CellCollector assay demonstrated a significant correlation between increasing positivity rates of IFCCs and advancing TNM stage, aligning with the positivity of carcinoembryonic antigen (CEA) detected by real‐time PCR. Notably, the positive rate for IFCCs identified by CellCollector was significantly higher than that of CEA via real‐time PCR, with global positivity rates of 32.9% and 51.4%, respectively.

**FIGURE 3:**
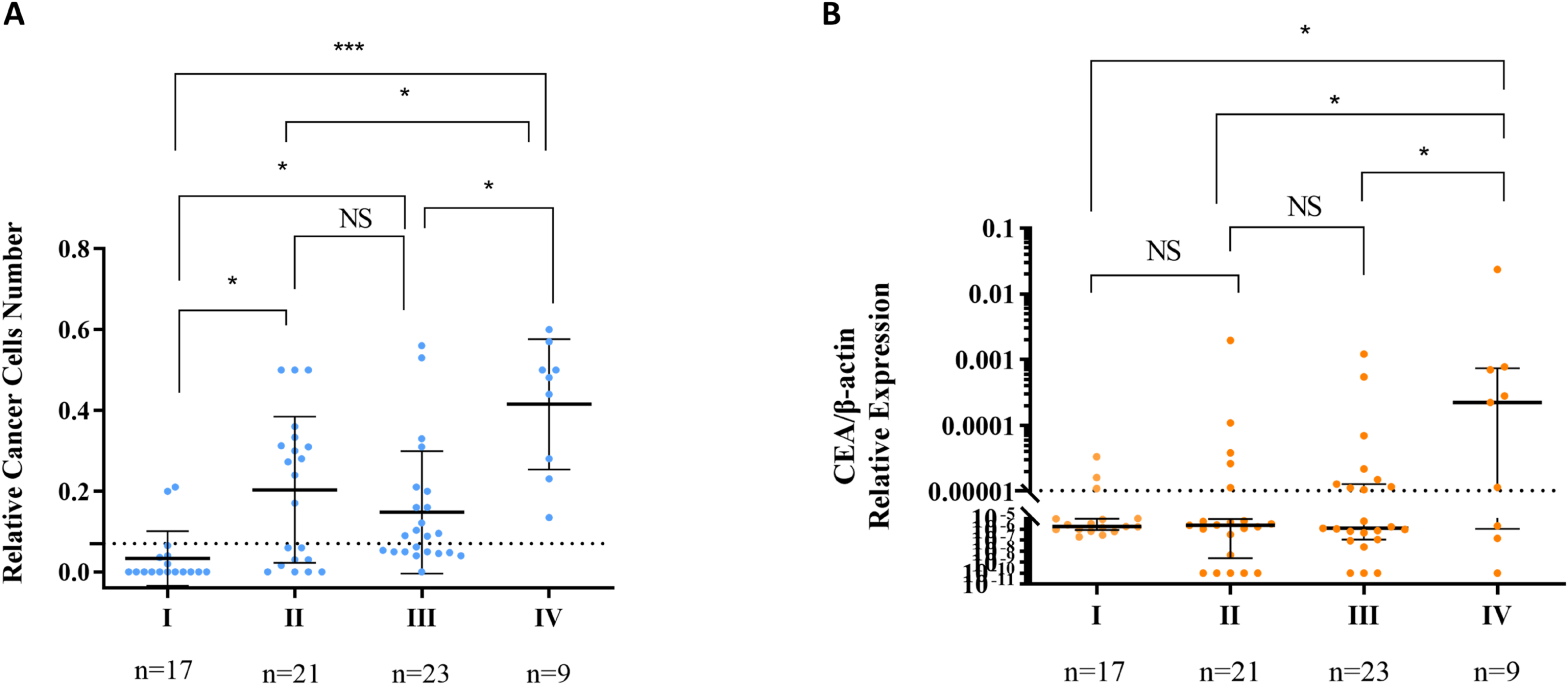
CellCollector and real‐time PCR assay detected the IFCCs in preoperative abdominal lavage fluid from different TNM stages CRC patients. (A) CellCollector assay in detection of the IFCCs in different TNM stages. Comparisons between each stage were significantly different, except for the difference in the positivity rate of IFCCs in patients with stages II and III, which was not statistically significant. (B) Real‐time PCR (CEA) assay in detecting the IFCCs in different TNM stages. The difference in the positivity rate of IFCCs between patients with stage I and II, and stages II and III was not statistically significant, but the differences between the other staging comparisons were significant. NS, no significant difference, *p < 0.05, **p < 0.01, ***p < 0.001.

As illustrated in Table 1 and Figure 3, CellCollector outperformed real‐time PCR in detecting IFCCs, particularly in stages II, III, and IV. Specifically, the positive rates for IFCCs detection using CellCollector were 57.1%, 56.4%, and 100.0% for stages II, III, and IV (the stage IV patients information could be seen in Table S4, and all the CRC patients TNM staging information could be seen in Table S5), respectively, compared to CEA's rates of 23.8%, 39.1%, and 66.7%. The overlap of T3 and T4 patients in stage II resulted in similar positivity rates for stages II and III. Among 47 patients deemed negative for IFCCs by real‐time PCR, 28 were also negative by CellCollector, while 19 were positive. Conversely, 17 out of 23 patients positive for IFCCs by real‐time PCR were confirmed positive by CellCollector.

**TABLE 1:** Relationship between the positivity rate of IFCCs (CellCollector and real‐time PCR assays) and clinical features in CRC patients.

Factor	All patients	CellCollector (positive rate)	*p*	Real‐time PCR (positive rate)	*p*
No. of patients	70	36 (51.4%)		23 (32.9%)	< 0.0001
Age (years)	56.7 ± 3.83				
Gender					
Male	54	29/54 (53.7%)	0.574	17/54 (31.5%)	0.258
Female	16	7/16 (43.8%)		6/16 (37.5%)	
Degree of differentiation					
G1–G2	48	27/48 (56.3%)	0.305	18/48 (37.5%)	0.656
G3–G4	22	9/22 (40.9%)		5/22 (22.7%)	
Primary tumor (T stage)					
T1–T2	18	3/18 (16.7%)	< 0.0001	4/18 (22.2%)	0.326
T3	25	11/25 (44.0%)		7/25 (28.0%)	
T4	27	22/27 (81.5%)		12/27 (44.4%)	
Regional lymph nodes (N stage)					
N−	38	14/38 (36.8%)	0.009	8/38 (21.1%)	0.04
N+	32	22/32 (68.8%)		15/32 (46.9%)	
Stage (TNM classification)					
I	17	2 (11.8%)	< 0.0001	3 (15.8%)	< 0.0001
II	21	12 (57.1%)		5 (23.8%)	
III	23	13 (56.5%)		9 (39.1%)	
IV	9	9 (100.0%)		6 (66.7%)	
Real‐time PCR evaluation					
Negative	47	19/47 (40.4%)	0.011		
Positive	23	17/23 (73.9%)			

**FIGURE 4:**
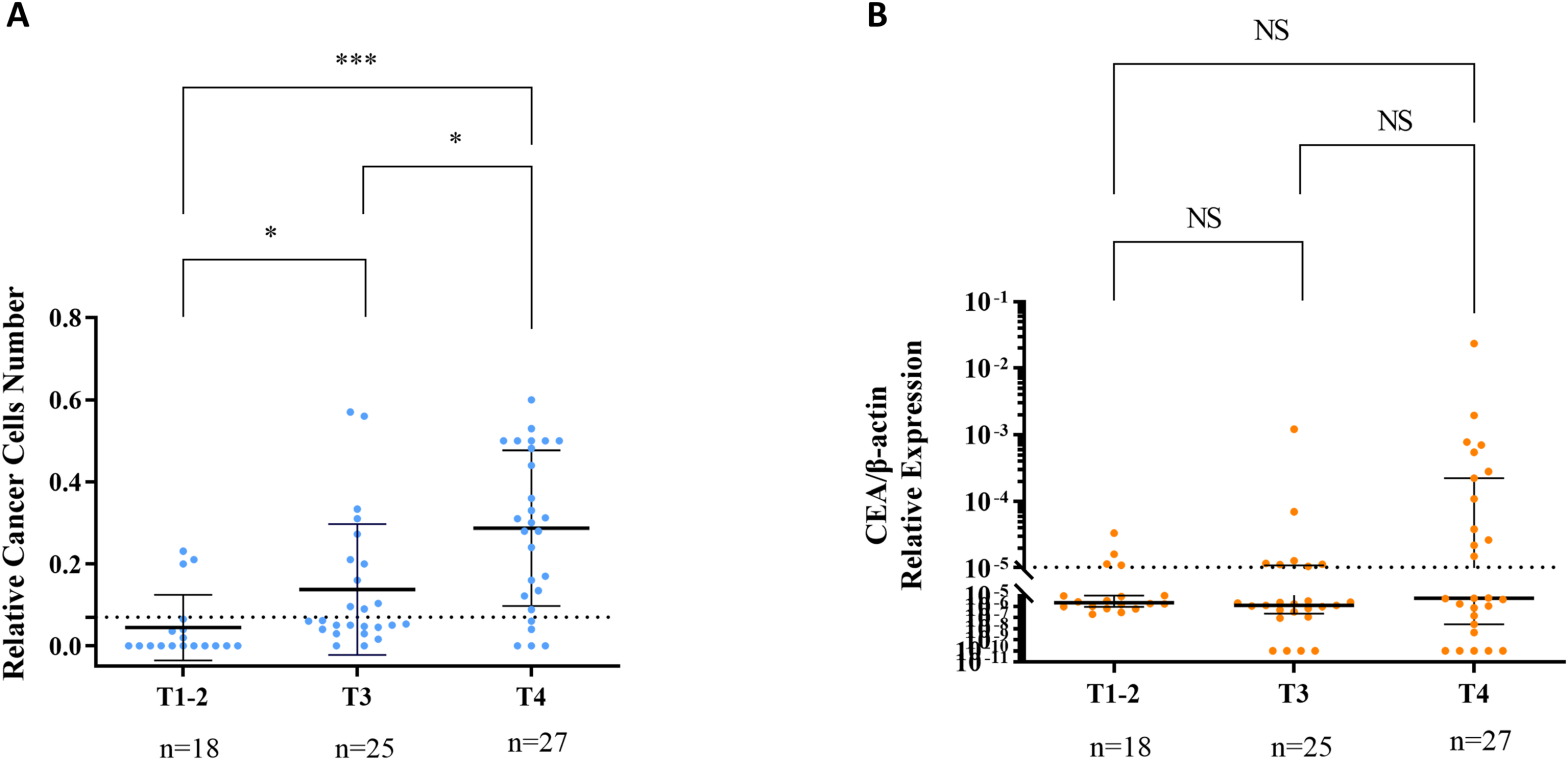
CellCollector and real‐time PCR assay detected the IFCCs in preoperative abdominal lavage fluid from different T stages of CRC patients. (A) CellCollector assay in detection of the IFCCs in different T stages. The differences in the positivity rates of IFCCs between each T stage were statistically significant. (B) Real‐time PCR (CEA) assay in the detection of the IFCCs in different T stages. None of the differences in the positivity rates of IFCCs between T stages were statistically significant. NS, no significant difference, *p < 0.05, ***p < 0.001.

Patients with T3‐T4 CRC are at increased risk for abdominal shedding of cancer cells due to tumor invasion of the serosal layer. Our analysis of T staging revealed that CellCollector identified IFCCs positivity rates of 44.0% and 81.5% for T3 and T4 stages, respectively, compared to only 28.0% and 44.4% for real‐time PCR (CEA). This underscores the superior ability of CellCollector in detecting IFCCs in advanced T stages, while real‐time PCR failed to distinguish positivity between T1‐2 and T3 patients (see Table 1 and Figure 4).

In the TNM staging system, lymph node metastasis (*N* stage) is crucial for determining stage III. Thus, *N* positivity significantly influences pathological staging and, consequently, IFCCs positivity rates in CRC patients. Our findings indicate that CellCollector exhibited a higher IFCCs positivity rate than real‐time PCR in N‐positive patients (Table 1).

Furthermore, as shown in Table 1, neither CellCollector nor real‐time PCR assays demonstrated any correlation with gender or tumor differentiation in detecting IFCCs positivity in CRC patients.

### 3.3 Differences in Preoperative and Postoperative Test Results

We assessed IFCCs in abdominal lavage fluid using both CellCollector and real‐time PCR assays at the commencement and conclusion of surgical procedures. Our findings revealed no significant difference in IFCCs detection by CellCollector before and after surgery. In contrast, real‐time PCR exhibited a significant increase in IFCCs positivity rates postoperatively compared to preoperatively.

**FIGURE 5:**
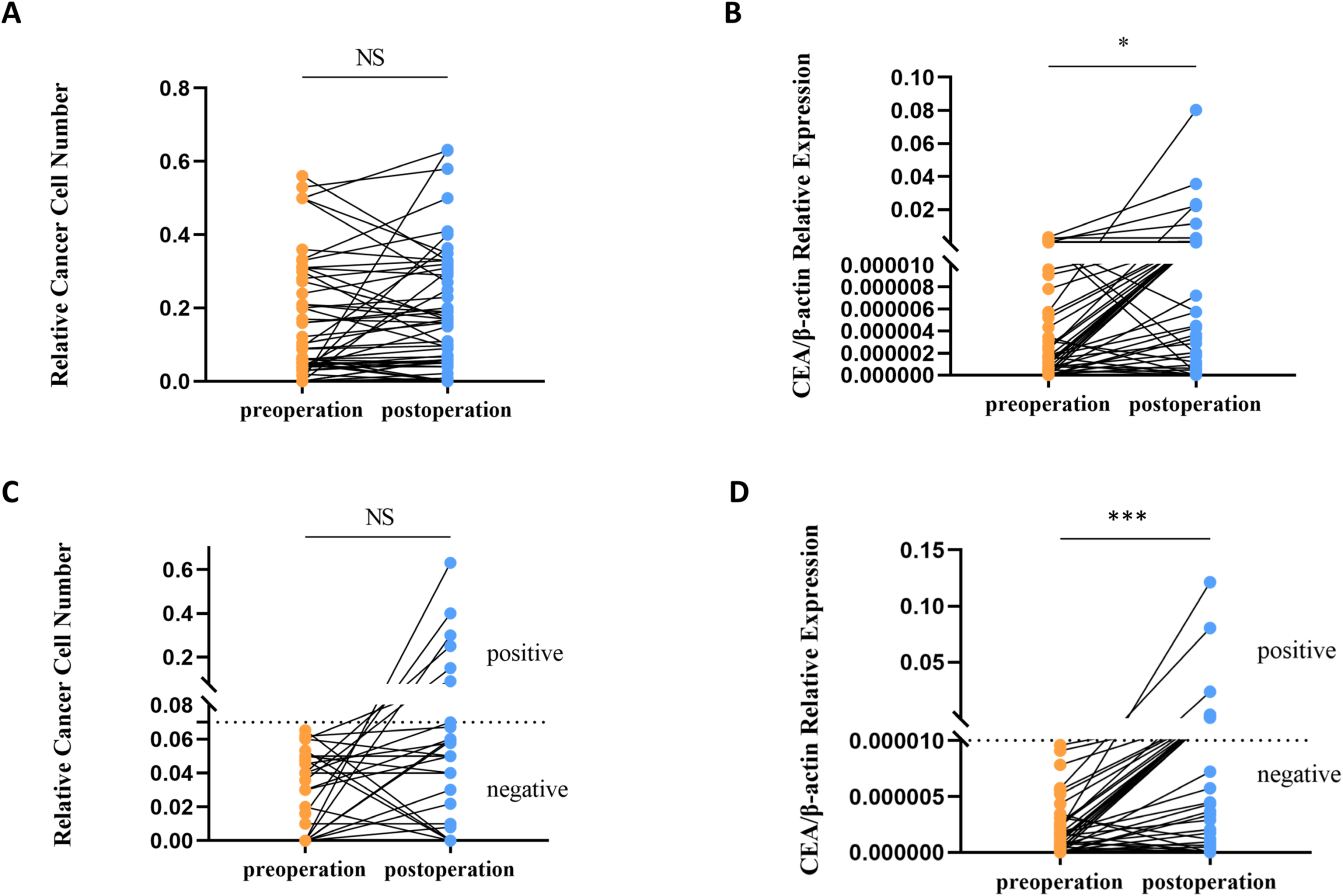
Results of preoperative and postoperative IFCCs testing in stage I–III CRC patients. (A) The difference between preoperative and postoperative IFCCs in stage I–III CRC patients detected by CellCollector assay was not statistically significant, *N* = 61. (B) Real‐time PCR (CEA) assay was statistically significant in detecting preoperative and postoperative IFCCs in stage I–III CRC patients, *N* = 61. (C) The difference between preoperative and postoperative IFCCs was not statistically significant in patients with stage I–III CRC who were preoperatively negative by CellCollector assay, *N* = 34. (D) Real‐time PCR (CEA) assay for preoperative negative stage I–III CRC patients showed a statistically significant difference between preoperative and postoperative IFCCs, *N* = 44. NS, no significant difference, *p < 0.05, ***p < 0.001.

To investigate potential medically induced cancer cell shedding during surgery, we analyzed the preoperative and postoperative results from patients who tested negative for IFCCs prior to surgery. The CellCollector assay showed no significant difference in IFCCs detection between preoperative and postoperative samples, while real‐time PCR indicated a marked difference (see Figure 5).

Given that the surgeries were conducted using strict TME or CME techniques, the likelihood of medically induced cancer cell shedding is minimal. Therefore, the consistent preoperative and postoperative IFCCs results suggest that the CellCollector assay may offer superior specificity and stability compared to real‐time PCR.

### 3.4 Bayesian Analysis of the Sensitivity and Specificity of Two Different Assays

We conducted a Bayesian analysis to evaluate the sensitivity and specificity of two assays based on predictions from cell line experiments and existing literature on CellCollector and tumor detection [26, 32, 33, 34, 35, 36, 37, 38]. The prior probabilities for CellCollector's sensitivity and specificity were set at 0.432–0.971 and 0.637–1.0, respectively. For real‐time PCR detecting CEA mRNA in CRC patients, the prior probabilities for sensitivity and specificity were established at 0.33–0.667 and 0.697–0.864, respectively [15, 16, 17, 27, 39, 40, 41, 42], The prior probability for the positive rate of IFCCs in CRC patients was set between 0.08 and 0.379, derived from relevant studies [15, 16, 17, 25, 27, 39, 40, 41, 42, 43].

Using the DTAXG package in R, we applied the Bayesian method to estimate sensitivity and specificity in the absence of a gold standard (Table 2). The results indicated that the sensitivity of CellCollector (84.6%) significantly exceeded that of real‐time PCR (48.4%) with a 95% confidence interval (95% CI, *p* < 0.0001). Additionally, CellCollector's specificity (79.2%) was higher than that of CEA (60.3%) with similar statistical significance (95% CI, *p* < 0.0001).

**TABLE 2:** Bayesian analysis of the sensitivity and specificity of CellCollector and real‐time PCR (CEA) assays.

	Positive rate	Se1	Se2	Sp1	Sp2
Median	0.3407817	0.8462424	0.4841107	0.7915927	0.6035508
2.5%	0.1641895	0.6267907	0.3457024	0.5841446	0.5163311
97.5%	0.5090183	0.9609751	0.6274390	0.9798553	0.6863583

*Note:* Se1: The sensitivity of CellCollector; Se2: The sensitivity of CEA mRNA; Sp1: The specificity of CellCollector; Sp2: The specificity of CEA mRNA.

## 4 Discussion

CRC ranks among the most prevalent cancers globally, with peritoneal carcinomatosis developing in 10%–20% of patients [44]. Numerous studies [6, 7, 10] have demonstrated that the presence of IFCCs correlates with CRC prognosis and can significantly inform intraoperative and postoperative management strategies. Preoperative peritoneal lavage serves as a valuable tool for detecting primary IFCCs, helping to assess the potential peritoneal spread of cancer cells. Similarly, identifying IFCCs in postoperative lavage can indicate surgical dissemination of cancer cells. The presence of positive IFCCs, both preoperatively and postoperatively, is associated with unfavorable prognostic outcomes and may necessitate more aggressive therapeutic interventions.

Peritoneal cytology detection has been used to detect the IFCCs in CRC [11, 21]. However, conventional cytology has significant limitations, primarily its low sensitivity (< 10% positive detection rate for CRC) and its high dependence on the operator's skill. These factors hinder the effectiveness of cytology in identifying IFCCs in CRC. Given the increasing importance of detecting IFCCs for therapeutic strategies in CRC, there is a pressing need for more sensitive and specific detection methods.

Guller et al. [19] introduced real‐time PCR as a more sensitive alternative for detecting IFCCs in CRC patients. In their study of 39 patients, they reported that 10 patients tested positive for IFCCs using real‐time PCR (targeting CEA and CK20) during peritoneal lavage. Notably, positive results from intraperitoneal real‐time PCR were identified as an independent prognostic factor for disease progression in eight patients who experienced recurrence during follow‐up. Similar findings were reported by Hara et al. [40] who noted that patients with positive real‐time PCR results for CEA had a poorer prognosis in CRC.

The use of real‐time PCR for detecting IFCCs in CRC patients presents several challenges. Some researchers have raised concerns that the expression of specific genes typically associated with cancer cells may also be found in inflammatory cells [45], resulting in real‐time PCR being a highly sensitive but low‐specific assay. In addition to mRNAs from IFCCs, real‐time PCR can inadvertently detect non‐cancer cell‐specific mRNA fragments. Furthermore, biomarkers such as CEA and CK20, which are commonly utilized for CRC detection, can be overexpressed in non‐cancerous cells under inflammatory conditions, complicating the interpretation of results.

The GILUPI CellCollector, an approved medical device, operates on the principle of utilizing antibodies against epithelial adhesion molecules (EpCAM) to capture tumor cells [26, 29]. Our experiments with cell lines demonstrated that the CellCollector exhibits high sensitivity and specificity. Notably, to our knowledge, there are currently no reports on the application of the CellCollector for detecting IFCCs in peritoneal lavage samples. This study represents the first instance of employing the CellCollector for IFCCs detection in CRC patients' peritoneal lavage. Additionally, we included real‐time PCR (CEA) as a control, and both methods displayed a higher positive detection rate.

Our results indicate an overall positive detection rate of 32.9% for IFCCs using the real‐time PCR (CEA) method, compared to 51.4% for the CellCollector assay. Both methods showed a positive correlation between IFCCs positivity and the TNM stage of CRC, with a statistically significant difference in overall positivity rates. Notably, the CellCollector demonstrated higher positivity rates for IFCCs than real‐time PCR in stages II, III, and IV CRC patients. In stage I patients, the positive rates for IFCCs detection were 11.8% for the CellCollector and 15.8% for real‐time PCR (CEA). Given that IFCCs are typically not expected in stage I patients, these findings suggest that both methods may exhibit high sensitivity but low specificity.

Bayesian analysis yielded sensitivity and specificity values of 0.846 and 0.792 for the CellCollector, and 0.484 and 0.604 for real‐time PCR, respectively. The ROC curve analysis revealed AUC of 0.876 for the CellCollector and 0.671 for real‐time PCR. These findings suggest that while the CellCollector is not a perfect method for detecting IFCCs, it demonstrates greater specificity compared to the commonly used real‐time PCR (CEA) method.

In the context of intra‐abdominal tumors, those that invade or breach the serosal layer are more likely to shed cells due to motion friction or intestinal peristalsis, potentially leading to a higher positivity rate for IFCCs [46]. Our subgroup analysis revealed that the positivity rate of the CellCollector assay in diagnosing IFCCs was higher than that of the real‐time PCR (CEA) method in CRC patients with T3 and T4 stages. This finding indicates that the CellCollector is more sensitive in capturing IFCCs than real‐time PCR.

Intraperitoneal lavage is primarily conducted to detect IFCCs in patients after abdominal opening and prior to any manipulation of the tumor. However, a limited number of studies have investigated the detection of IFCCs before and after tumor resection. Two studies employing real‐time PCR reported preoperative detection rates of 12% and 14%, but they exhibited significant variability in postoperative detection rates—one study reported a decrease to 3%, while the other found an increase to 20% [17, 21]. These discrepancies may stem from assay instability or variability in surgical techniques. To address this controversy, our study implemented rigorous quality control measures during surgical procedures to minimize the shedding of cancer cells. We standardized surgeries according to TME or CME protocols and adhered to the principle of avoiding direct contact with the tumor during surgery. The only variable was the detection methods applied to preoperative and postoperative abdominal lavage fluids for IFCCs detection. The results demonstrated statistically significant differences in IFCCs detection between preoperative and postoperative samples using the real‐time PCR assay. In contrast, the CellCollector assay did not show significant differences between preoperative and postoperative samples. This suggests that the CellCollector may provide a more stable and reliable method for detecting IFCCs compared to the real‐time PCR (CEA) assay.

## 5 Conclusions

Our findings indicate that the CellCollector offers distinct advantages over real‐time PCR in the detection of IFCCs in CRC, demonstrating higher sensitivity and specificity. However, this study has certain limitations, including a relatively small sample size and the higher cost associated with the CellCollector testing. To further validate the sensitivity and specificity of the CellCollector and facilitate its clinical application, it is essential to increase the sample size in future studies.

Additionally, conducting a follow‐up analysis of the patients involved in this study is crucial for comparing the short‐ and long‐term survival rates between IFCC‐positive and IFCC‐negative patients. Such analysis could provide valuable insights for guiding subsequent treatment decisions and improving patient management in CRC.

## Author Contributions

**Yudi Wu:** conceptualization (equal), data curation (equal), methodology (equal), writing – original draft (equal). **Fangxun He:** methodology (equal), software (equal). **Liang Liu:** data curation (equal), resources (equal), validation (equal), visualization (equal). **Wei Jiang:** data curation (equal), investigation (equal), resources (equal). **Jiao Deng:** methodology (equal). **Yujie Zhang:** formal analysis (equal), software (equal). **Zhixin Cao:** supervision (equal). **Xiangshang Xu:** conceptualization (equal), funding acquisition (equal), methodology (equal), project administration (equal), writing – review and editing (equal). **Jianping Gong:** conceptualization (equal), funding acquisition (equal), supervision (equal).

## Ethics Statement

All procedures performed in studies involving human participants followed the institutional and/or national research committee's ethical standards, the 1964 Helsinki Declaration, and its later amendments or comparable ethical standards. The study was approved by the ethical committee of Tongji Hospital, Tongji Medical College, HUST (TJ‐IRB20230215). All methods were performed under the relevant guidelines and regulations.

## Consent

The authors confirm: the work described has not been published before; it is not under consideration for publication elsewhere; all co‐authors have approved its publication.

## Conflicts of Interest

The authors declare no conflicts of interest.

## Supporting information


Tables S1–S5.

## Data Availability

The analyzed data sets generated during the study are available from the corresponding author on reasonable request.
