# Supplementary material for: The Use of CellCollector Assay to Detect Free Cancer Cells in the Peritoneal Cavity of Colorectal Cancer Patients: An Experimental Study
Source: Cancer Med. 2024 Nov 6;13(21):e70378. doi: 10.1002/cam4.70378 (PMC11538901; doi:10.1002/cam4.70378)
Supplement: Supplementary file 1 — Tables S1–S5. [file CAM4-13-e70378-s001.docx]

**Supplementary Table 1.** Primers Sequence

| Name | Primer | Tm (℃) | product length |
| --- | --- | --- | --- |
| β-actin primer forward | CCTGTACGCCAACACAGTGC | 61.84 | 211 |
| β-actin primer reverse | ATACTCCTGCTTGCTGATCC | 57.07 |  |
| CEA primer forward | GACGCAAGAGCCTATGTATG | 56.12 | 270 |
| CEA primer reverse | GGCATAGGTCCCGTTATTA | 53.68 |  |

**Supplementary Table 2.** Pathological information of patients whose IFCCs results were used to draw the ROC curve

| Patients with Benign disease | Clinical diagnosis | Patients with CRC | Pathological stage |
| --- | --- | --- | --- |
| 1 | Appendicitis | 1 | T4aN0M0 |
| 2 | Appendicitis | 2 | T4aN0M0 |
| 3 | Appendicitis | 3 | T4aN0M0 |
| 4 | Gastric perforation | 4 | cT4bNxM1 |
| 5 | Gastric perforation | 5 | T4aN1M0 |
| 6 | Appendicitis | 6 | cT4bNxM1 |
| 7 | Gastric perforation | 7 | T4aN2M0 |
| 8 | Gastric perforation | 8 | T4aN0M0 |
| 9 | Appendicitis | 9 | cT4bNxM1 |
| 10 | Appendicitis | 10 | T4aN1M0 |
| 11 | Appendicitis | 11 | T4aN0M1 |
| 12 | Gastric perforation | 12 | T4bN0M0 |
| 13 | Appendicitis | 13 | T4bN0M0 |
| 14 | Appendicitis | 14 | T4bN0M0 |
| 15 | Appendicitis | 15 | T4aN0M0 |
| 16 | Gastric perforation | 16 | T4aN0M0 |
| 17 | Appendicitis | 17 | T4aN0M0 |
|  |  | 18 | T4bN1M1 |
|  |  | 19 | T4aN0M0 |
|  |  | 20 | T4aN1M0 |
|  |  | 21 | T4aN0M0 |
|  |  | 22 | T4aN2M1 |
|  |  | 23 | T4bN0M1 |
|  |  | 24 | T4aN1M1 |
|  |  | 25 | T4aN0M0 |
|  |  | 26 | T4aN1M0 |
|  |  | 27 | cT4bNxM1 |

**Supplementary Table 3.** Normal epithelial cells and positive cells captured by CellCollector assay in abdominal lavage fluid from clinical patients in the pre-test.

| Cells/Patients No. | 1 | 2 | 3 | 4 | 5 | 6 | 7 | 8 | 9 |
| --- | --- | --- | --- | --- | --- | --- | --- | --- | --- |
| Normal epithelial cells | 9 | 19 | 26 | 4 | 5 | 16 | 15 | 9 | 9 |
| Positive cells | 0 | 0 | 1 | 0 | 1 | 3 | 4 | 1 | 2 |

No.1-4: patients with benign disease; No.5-9: patients with colorectal cancer.

**Supplementary Table 4.** Pathological information of patients with stage IV colorectal cancer

| Patients | Pathological stage | Sites of metastasis |
| --- | --- | --- |
| 1 | cT4bNxM1 | Peritoneum |
| 2 | cT4bNxM1 | Peritoneum |
| 3 | cT4bNxM1 | Peritoneum |
| 4 | T4aN0M1 | Peritoneum |
| 5 | T4bN1M1 | Liver |
| 6 | T4aN2M1 | Liver |
| 7 | T4bN0M1 | Peritoneum |
| 8 | T4aN1M1 | Liver |
| 9 | cT4bNxM1 | Peritoneum |

**Supplementary Table 5.** Pathological information of all patients with colorectal cancer

| Patients | Pathological stage | TNM stagings | Sites of tumor |
| --- | --- | --- | --- |
| 1 | cT4bNxM1 | IV | Rectum |
| 2 | T4aN0M0 | II | Rectum |
| 3 | T4aN0M0 | II | Descending colon |
| 4 | T4aN0M0 | II | Rectum |
| 5 | T2N0M0 | I | Rectum |
| 6 | T3N2M0 | III | Ascending colon |
| 7 | T3N2M0 | III | Sigmoid colon |
| 8 | T2N0M0 | I | Rectum |
| 9 | T2N0M0 | I | Rectum |
| 10 | cT4bNxM1 | IV | Sigmoid colon |
| 11 | T2N0M0 | I | Sigmoid colon |
| 12 | T3N1M0 | III | Ascending colon |
| 13 | T4aN1M0 | III | Rectum |
| 14 | cT4bNxM1 | IV | Ascending colon |
| 15 | T4aN2M0 | III | Rectum |
| 16 | T4aN0M0 | II | Rectum |
| 17 | T3N0M0 | II | Sigmoid colon |
| 18 | T4aN0M1 | IV | Sigmoid colon |
| 19 | T3N2M0 | III | Rectum |
| 20 | T4aN1M0 | III | Sigmoid colon |
| 21 | T3N1M0 | III | Rectum |
| 22 | T3N0M0 | II | Sigmoid colon |
| 23 | T3N1M0 | III | Sigmoid colon |
| 24 | T4bN1M1 | IV | Rectum |
| 25 | T3N0M0 | II | Sigmoid colon |
| 26 | T2N0M0 | I | Sigmoid colon |
| 27 | T4bN0M0 | II | Ascending colon |
| 28 | T2N0M0 | I | Descending colon |
| 29 | T4bN0M0 | II | Sigmoid colon |
| 30 | T3N1M0 | III | Rectum |
| 31 | T2N0M0 | I | Rectum |
| 32 | T4bN0M0 | II | Descending colon |
| 33 | T3N1M0 | III | Sigmoid colon |
| 34 | T3N0M0 | II | Rectum |
| 35 | T3N1M0 | III | Sigmoid colon |
| 36 | T3N1M0 | III | Rectum |
| 37 | T4aN0M0 | II | Ascending colon |
| 38 | T1bN0M0 | I | Ascending colon |
| 39 | T3N0M0 | II | Rectum |
| 40 | T4aN0M0 | II | Rectum |
| 41 | T3N2M0 | III | Ascending colon |
| 42 | T2N0M0 | I | Ascending colon |
| 43 | T4N0M0 | II | Rectum |
| 44 | T4N1M0 | III | Sigmoid colon |
| 45 | T4aN0M0 | II | Sigmoid colon |
| 46 | T4aN1M0 | III | Ascending colon |
| 47 | T3N2M0 | III | Sigmoid colon |
| 48 | T4aN0M0 | II | Rectum |
| 49 | T2N0M0 | I | Rectum |
| 50 | T3N1M0 | III | Sigmoid colon |
| 51 | T3N1M0 | III | Ascending colon |
| 52 | T2N0M0 | I | Sigmoid colon |
| 53 | T3N0M0 | II | Rectum |
| 54 | T3N0M0 | II | Rectum |
| 55 | T2N0M0 | I | Rectum |
| 56 | T2N0M0 | I | Ascending colon |
| 57 | T3N1M0 | III | Sigmoid colon |
| 58 | T4aN2M1 | IV | Ascending colon |
| 59 | T4bN0M1 | IV | Ascending colon |
| 60 | T4aN1M1 | IV | Rectum |
| 61 | T2N0M0 | I | Rectum |
| 62 | cT4bNxM1 | IV | Rectum |
| 63 | T4aN0M0 | II | Descending colon |
| 64 | T4aN1M0 | III | Sigmoid colon |
| 65 | T3N1M0 | III | Sigmoid colon |
| 66 | T2N0M0 | I | Rectum |
| 67 | T3N0M0 | II | Ascending colon |
| 68 | T4aN1M0 | III | Rectum |
| 69 | T2N0M0 | I | Ascending colon |
| 70 | T2N0M0 | I | Sigmoid colon |
